# FBXW7 gene polymorphism is associated with type 2 diabetes in the Uygur population in Xinjiang, China

**DOI:** 10.1186/s41065-021-00191-z

**Published:** 2021-08-09

**Authors:** Shi-Qi Yan, Dilare Adi, Cheng Liu, Meng-Meng Wang, Jialin Abuzhalihana, Yun Wu, Zhen-Yan Fu, Yi-Ning Yang, Xiao-Mei Li, Xiang Xie, Fen Liu, Bang-Dang Chen, Yi-Tong Ma

**Affiliations:** 1 Department of Cardiology, First Affiliated Hospital of Xinjiang Medical University, Urumqi, 830054 P.R. China; 2 Xinjiang Key Laboratory of Cardiovascular Disease Research, Urumqi, 830054 P.R. China

**Keywords:** FBXW7, Polymorphism, Type 2 diabetes

## Abstract

**Background:**

FBXW7 gene expression is positively correlated with glycolipid metabolism and is associated with diabetes in animal models. In the current study, we focused on exploring whether genetic variants of the FBXW7 gene were associated with type 2 diabetes (T2DM) and the risk factors for T2DM in Uygur people in Xinjiang, China.

**Methods:**

A total of 2164 Chinese Uygur subjects (673 T2DM patients and 1491 controls) were recruited for our case–control study, and four SNPs (rs10033601, rs2255137, rs2292743 and rs35311955) of the FBXW7 gene were genotyped using the improved multiplex ligation detection reaction (iMLDR) technique.

**Results:**

Our study showed that the genotypes using the overdominant model (GA vs AA + GG) of rs10033601 and using the overdominant model (TA vs TT + AA) of rs2292743 were significantly different between T2DM patients and the controls (*P* = 0.005 and *P* = 0.012, respectively). After multivariate adjustments for confounders, the rs10033601 and rs2292743 SNPs were still independent risk factors for T2DM [GA vs AA + GG: odds ratio = 1.35, 95% confidence interval (CI) = 1.12–1.64, *P* = 0.002; TA vs TT + AA: OR = 1.28, 95% CI = 1.06–1.55, *P* = 0.011]. Participants within the Chinese Uygur populations and who with the GA genotype of rs10033601 and the TA genotype of rs2292743 were associated with significantly elevated glucose levels.

**Conclusions:**

Our study revealed that both rs10033601 and rs2292743 of the FBXW7 gene were associated with T2DM in the Uygur populations in Xinjiang.

## Introduction

Type 2 diabetes mellitus (T2DM), which is the most common and dominant type of diabetes and accounts for approximately 90% of all cases, is a multifactorial disorder with an increasing incidence [1]. A large number of risk factors are associated with T2DM, including lifestyle changes, environmental factors and genetic components [2–5]. T2DM is the most common endocrine and metabolic disease, is caused by a combination of environmental and genetic factors and is also a recognized independent risk factor for cardiovascular disease (CAD) [6]. Persistently high blood glucose levels can lead to micro- and macrovascular damage, which further leads to CAD [7]. In recent decades, studies have demonstrated the polygenic aspect of T2DM and have revealed the effects of numerous gene variants on the susceptibility of the disease [8–11].

F-box and WD repeat domain-containing 7 (FBXW7) is an E3-ubiquitin ligase that mediates the recognition of phosphorylated substrates, such as SREBPs, for proteolysis [12, 13]. FBXW7 interacts with nuclear SREBP family genes and improves their ubiquitination, eventually resulting in their degradation, suggesting that FBXW7 could regulate blood lipids [12–16]. Several studies have demonstrated that disorders of lipid metabolism are involved in the pathogenesis of CAD [17, 18]. Our preliminary findings suggested that the FBXW7 gene variant is associated with CAD in the Uygur Chinese population. Interestingly, Fbxw7 has also been noted to play a vital role in glucose homeostasis by promoting the ubiquitination and degradation of Fetuin-A in a manner dependent on the phosphorylation of Serine 305 and Serine 309. Liver-specific ablation of FBXW7 disrupts glucose homeostasis. However, in the obese liver, FBXW7 expression confers several beneficial metabolic effects, such as protection against glucose intolerance, insulin resistance, and hyperglycaemia [19]. Additionally, despite the widespread reports of Fbxw7 involvement in glucose metabolism, its single nucleotide polymorphisms (SNPs) have not been studied. Hence, the aim and focus of the present research was to assess the associations between genetic polymorphisms of FBXW7 and T2DM in the Uygur Chinese populations in Xinjiang, China.

## Methods

### Ethical approval of the study protocol

The Ethics Committee of the First Affiliated Hospital of Xinjiang Medical University approved this study (Ethical approval number: K202011-06), and it was executed in accordance with the standards of the Declaration of Helsinki. Each participant gave written informed consent and explicit permission for pertinent clinical data collection and DNA analyses.

### Subjects

A total of 2164 Uygur participants were recruited randomly (1483 men, 681 women) from the Medical University Hospital between August 2013 and November 2016. These subjects, who all resided in the Xinjiang Uygur Autonomous Region of China, passed the eligibility criteria and had genotyping data of the FBXW7 gene. A total of 672 patients that were diagnosed with T2DM were assigned to the case group. T2DM was defined as those who had a fasting plasma glucose (FPG) ≥ 7.0 mmol/l, a 2 h postload plasma glucose (2 hPG) ≥ 11.1 mmol/l, or a history of having hypoglycaemia therapy [20]. A total of 1492 subjects with normal glucose tolerance (NGT) were designated as the control group. NGT was defined as FPG < 6.1 mmol/l and 2 hPG < 7.8 mmol/l.

### Genotyping

**Fig. 1:**
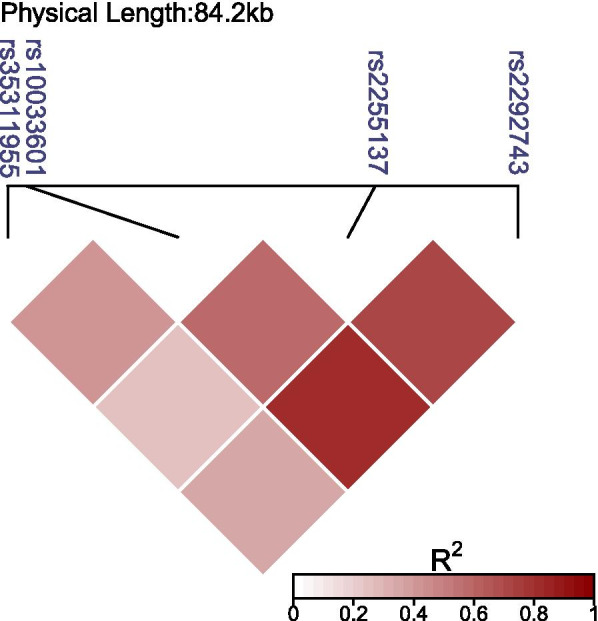
Genetic variation of the human Fbxw7 gene. Using the Ldheatmap software in R package. we scanned four genotyped single-nucleotide polymorphisms (SNPs) in Chinese Uygur subjects. Linkage disequilibrium (LD) blocks across this locus in Chinese Uygur subjects are shown. The LD values are displayed as follows: the higher the*R*2 value, the deeper the redness of the color

Four tag SNPs of FBXW7 were obtained using Haploview 4.2 software alongside the International HapMap project website phases I and II (http://www.hapmap.org). rs10033601 (SNP1), rs2255137 (SNP2), rs2292743 (SNP3), and rs35311955 (SNP4) were included by using the minor allele frequency (MAF) ≥ 0.05 and the linkage disequilibrium patterns with r^2^ ≥ 0.8 as the cut-off. We perform the linkage disequilibrium blocks in the genome area in FBXW7 genes by using the Ldheatmap software in R package. As shown in Fig. 1, the LD values are displayed as follows: the higher the *R*^2^ value, the deeper the redness of the color. A standard venipuncture technique was used to obtain blood samples from the participants using EDTA-containing tubes. Using a blood genome extraction kit, we extracted the DNA from the blood leukocytes at the periphery (Beijing Bioteke Corporation, Beijing, China). Without knowledge of the subject’s data, genotyping was performed, and approximately 10% of the samples were duplicated for the purposes of monitoring the quality of the genotyping.

### Statistical analysis

SPSS version 23.0 for Windows was used to perform the statistical analyses of the study (SPSS Inc. Chicago, IL, USA). The chi-square test was used to assess the equilibrium assessed by the Hardy–Weinberg test. The Kolmogorov–Smirnov test was used to examine the normality of the variables. Data were reported as the mean plus or minus the S.D. for normally distributed variables. However, the difference between the male and female patients was assessed with the two-independent-sample T-test. The chi-square test was used to analyse the categorical data, such as the differences of the frequencies of drinking, genotypes, smoking and hypertension between the groups. General linear analysis was performed to test the existing association between the lipid profiles and the FBXW7 genotypes, and logistic regression analyses were used to assess the contribution of the major risk factors. Two-tailed *P*-values of 0.05 were considered significant.

## Results

### Clinical characteristics of subjects

The subjects’ baseline characteristics are shown in Table 1. There were significant differences in serum total cholesterol (TC), triglycerides (TGs), low-density lipoprotein cholesterol (LDL-C), high-density lipoprotein cholesterol (HDL-C), fasting blood glucose (FBG), body mass index (BMI), blood urea nitrogen (Bun), uric acid levels and prevalence of hypertension between the two groups (all *P* < 0.05).

**Table 1:** Clinical and metabolic characteristics of subjects

**Characteristics**	**Control (*n* = 1491)**	**DM (*n* = 673)**	**χ2 or t**	***P* value**
Age (years)	56.9 ± 11.8	57.8 ± 11.9	2.013	0.052
Gender, female (%)	465 (31.2%)	216 (32.1%)	0.177	0.689
Smoking, n(%)	787 (52.8%)	377 (56%)	1.952	0.177
Drinking, n (%)	473 (31.7%)	217 (31.2%)	0.058	0.842
Hypertension, n (%)	714 (47.6%)	448 (66.6%)	65.079	** < 0.001**
BMI, mean (SD)	25.84 ± 3.41	26.17 ± 3.39	2.058	**0.040**
Cr, mean (SD)	73.80 ± 18.03	73.43 ± 20.27	0.425	0.671
BUN, mean (SD)	5.38 ± 1.46	5.56 ± 2.05	2.356	**0.019**
Uric acid, mean (SD)	318.65 ± 81.47	302.41 ± 81.90	4.284	** < 0.001**
FBG, mean (SD)	5.18 ± 1.29	8.08 ± 3.66	27.060	** < 0.001**
TG, mean (SD)	1.79 ± 1.11	2.00 ± 1.29	3.918	** < 0.001**
TC, mean (SD)	4.11 ± 1.22	3.98 ± 1.27	2.206	**0.028**
HDL-C, mean (SD)	1.06 ± 0.33	0.98 ± 0.31	4.985	** < 0.001**
LDL-C, mean (SD)	2.55 ± 0.86	2.41 ± 0.97	3.465	** < 0.001**

Data are presented as number of patients (%) or mean standard ± deviation

*BMI* Body mass index, *Cr* Creatinine, *BUN* Blood urea nitrogen, *FBG* Fasting blood glucose, *TG* Triglyceride, *TC* Total cholesterol, *HDL-C* High density lipoprotein-cholesterol, *LDL-C* Low-density lipopretein-cholesterol

### Distribution of SNPs of FXBW7 gene in subjects

The distributions of the SNPs in the FXBW7 gene are shown in Table 2. The genotype distributions of the four SNPs were in accordance with the Hardy–Weinberg equilibrium balance (all *P* > 0.05). Our results indicated that the distributions of rs2255137 and rs35311955 SNPs between the case and control groups were similar (all *P* > 0.05). For rs10033601 and rs2292743, the distribution of genotypes and the overdominant model{(GA vs AA + GG), (TA vs TT + AA)}showed a significant difference between the two groups (*P* = 0.005, *P* = 0.012).

**Table 2:** Distribution of SNPs of FXBW7 gene in the cases and controls

**Genotype**	**Model**		**Case (n, %)**	**Control (n, %)**	***P* value**
rs10033601	Codominant	AA	254 (37.7)	512 (34.3)	**0.018**
		GA	281 (41.8)	719 (48.2)	
		GG	138 (20.5)	260 (17.4)	
	Dominant	AA	254 (37.7)	512 (34.3)	0.132
		GA + GG	419 (62.3)	979 (65.7)	
	Recessive	AA + GA	535 (79.5)	1231 (82.6)	0.093
		GG	138 (20.5)	260 (17.4)	
	Overdominant	AA + GG	392 (58.2)	772 (51.8)	**0.005**
		GA	281 (41.8)	719 (48.2)	
	Allele	A	789 (58.6)	1743 (58.5)	0.918
		G	557 (41.4)	1239 (41.5)	
rs2255137	Codominant	TT	199 (29.6)	426 (28.6)	0.471
		CT	311 (46.2)	730 (49.0)	
		CC	163 (24.2)	335 (22.5)	
	Dominant	TT	199 (29.6)	426 (28.6)	0.335
		CT + CC	474 (70.4)	1065 (71.4)	
	Recessive	TT + CT	510 (75.8)	1156 (77.5)	0.37
		CC	163 (24.2)	335 (22.5)	
	Overdominant	TT + CC	362 (53.8)	761 (51.0)	0.236
		CT	311 (46.2)	730 (49.0)	
	Allele	T	709 (52.7)	1582 (53.1)	0.818
		C	637 (42.3)	1400 (46.9)	
rs2292743	Codominant	TT	158 (23.5)	318 (21.3)	**0.039**
		TA	288 (42.3)	726 (48.7)	
		AA	227 (33.7)	447 (30.0)	
	Dominant	TT	158 (23.5)	318 (21.3)	0.263
		TA + AA	515 (76.5)	1173 (78.7)	
	Recessive	TT + TA	446 (66.3)	1044 (70.0)	0.088
		AA	227 (33.7)	447 (30.0)	
	Overdominant	TT + AA	385 (57.2)	765 (51.3)	**0.012**
		TA	288 (42.8)	726 (48.7)	
	Allele	T	604 (44.9)	1362 (45.7)	0.624
		A	742 (55.1)	1620 (54.3)	
rs35311955	Codominant	GG	400 (59.4)	886 (59.4)	0.607
		GC	230 (34.2)	525 (35.2)	
		CC	43 (6.4)	80 (5.4)	
	Dominant	GG	400 (59.4)	886 (59.4)	1.000
		GC + CC	273 (40.6)	606 (40.6)	
	Recessive	GG + GC	630 (93.6)	1411 (94.6)	0.367
		CC	43 (6.4)	80 (5.4)	
	Overdominant	GG + CC	360 (53.7)	758 (51.0)	0.712
		GC	230 (34.2)	728 (49.0)	
	Allele	G	1030 (76.5)	2297 (77.0)	0.715
			316 (23.5)	685 (23.0)	

Multivariable logistic regression analyses revealed that after adjusting for the major confounding factors, including BMI, BUN, TG, uric acid, Cr, LDL-C, age, TC, sex, HDL-C, smoking, and hypertension, the rs10033601 SNP was an independent risk factor for T2DM [GA vs AA + GG: odds ratio (OR) = 1.35, 95% confidence interval (CI) = 1.12–1.64, *P* = 0.002, Table 3]. However, after adjusting for confounders, rs2292743 was still an independent risk factor for T2DM [TA vs TT + AA: OR = 1.28, 95% CI = 1.06–1.55, *P* = 0.011, Table 4].

**Table 3:** Results of logistic analysis (rs10033601)

	**OR**	**95%CI**	***P* value**
rs10033601(GA vs.AA + GG)	1.354	1.118–1.640	**0.002**
Gender	1.142	0.872–1.495	0.333
Age	1.012	1.001–1.024	0.032
BMI	1.024	0.995–1.053	0.110
Hypertension	2.059	1.689–2.510	**0.000**
Smoking	1.242	0.972–1.588	**0.084**
Drinking	1.009	0.791–1.286	0.943
BUN	1.091	1.027–1.158	0.005
Cr	1.001	0.995–1.007	0.720
Uric acid	0.996	0.995–0.997	0.000
TG	1.181	1.084–1.286	0.000
TC	0.934	0.832–1.049	0.250
HDL-C	0.507	0.364–0.706	0.000
LDL-C	0.909	0.776–1.064	0.234

**Table 4:** Results of logistic analysis (rs2292743)

	**OR**	**95%CI**	***P* value**
rs2292743(TA vs.TT + AA)	1.280	1.058–1.550	**0.011**
Gender	1.143	0.873–1.496	0.331
Age	1.012	1.001–1.023	**0.037**
BMI	1.023	0.995–1.053	0.113
Hypertension	2.045	1.078–2.493	**0.000**
Smoking	1.248	0.997–1.595	0.076
Drinking	1.000	0.785–1.274	0.999
BUN	1.092	1.028–1.160	**0.004**
Cr	1.001	0.995–1.007	0.713
Uric acid	0.996	0.995–0.997	**0.000**
TG	1.183	1.086–1.288	**0.000**
TC	0.935	0.832–1.050	0.254
HDL-C	0.511	0.367–0.712	**0.000**
LDL-C	0.912	0.779–1.068	0.253

### Univariate logistic regression analysis of rs10033601 and rs2292743 genotypes for T2DM risk factors

The relationship between FXBW7 gene polymorphisms and recognized risk factors for T2DM as following in Tables 5 and 6. The rs2292743 SNPs were shown to significantly increase T2DM risk in obese people with BMI ≥ 28 kg/m^2^ (TA vs TT + AA: OR = 1.37, 95% CI = 1.13–1.67, *P* = 0.002).

**Table 5:** Univariate logistic regression analysis of rs10033601 genotypes for T2DM risk factors

rs10033601	AA + GG (*n* = 1164)	GA (*n* = 1000)	OR	95%CI	*P*
BMI ≥ 28 kg/m2	319 (26.9%)	237 (23.7%)	1.184	0.974–1.439	0.089
Age ≥ 48	1001 (86.0%)	841 (84.1%)	1.161	0.916–1.471	0.217

**Table 6:** Univariate logistic regression analysis of rs2292743 genotypes for T2DM risk factors

rs2292743	TT + AA (*n* = 803)	TA (*n* = 1014)	OR	95%CI	*P*
BMI ≥ 28 kg/m2	324 (28.2%)	226 (22.3%)	1.368	1.124–1.664	**0.002**
Age ≥ 48	995 (86.5%)	847 (83.5%)	1.266	0.999–1.604	0.051

## Discussion

In the present study, we found that rs10033601 and rs2292743 variations in the FBXW7 gene were all strongly correlated with the T2DM susceptibility in the Uygur Chinese population. Thus far, this was the first study to investigate the common allelic variants in the FBXW7 gene and their association with T2DM in the Uygur Chinese population.

Previous investigations have revealed the associations of the FBXW7 gene with tumour suppression and the progression of the cell cycle [21–23]. Moreover, FBXW7 has an essential role in regulating the metabolism of energy and lipids. FBXW7 regulates the functions of the SREBO protein family that control the synthesis of triglycerides and their stability. SREBP family gene stabilization enhances cholesterol and fatty acid synthesis and induces the expression of SREBP alongside the uptake of LDL-C [24]. In addition, Bengoechea et al. [25] found that FBXW7 negatively regulates adipocyte differentiation through targeted intervention in C/EBP α for degradation. FBXW7 plays key roles in regulating lipogenesis and cell proliferation and differentiation in the liver, and this has been shown both in basic biological and clinical experiments.

Alteration of lipid metabolism is a risk factor and characteristic feature of atherosclerosis. Atherosclerosis develops as a result of a multistep process ultimately leading to cardiovascular disease associated with a high morbidity and a high mortality. As previously mentioned, T2DM is a metabolic disorder characterized by chronic hyperglycaemia. Changes in blood lipid levels in diabetic patients are associated with an increased production of triglyceride-rich lipoproteins in the liver, leading to an increased formation of atherosclerotic very low-density lipoproteins (VLDL) [26]. This can eventually accelerate the development of atherosclerosis and even lead to CAD.

The relationship between T2DM and Fbxw7 was first studied by Zhao et al. [27]; they investigated the liver triglyceride contents as well as the expression levels of proinflammatory cytokines in WAT of FBXW7 LKO mice and found that they were markedly increased. Additionally, this study knocked down fetuin-A expression in FBXW7 LKO mice and observed that FBXW7 improves glucose metabolism by promoting fetuin-A degradation. A corresponding decrease in fetuin-A levels was also observed in FBXW7-overexpressing obese mice compared to WT controls. These findings showed that FBXW7 contributed to the elevated glucose levels.

In the present study, we genotyped polymorphisms of rs10033601, rs2255137, rs2292743 and rs35311955 of SNPs in the FBXW7 gene and found that rs10033601 and rs2292743 were associated with T2DM. The rs10033601 GA genotype and the rs2292743 TA genotype had higher frequencies in T2DM patients than in controls. After adjusting for several confounders, this association remained, indicating that rs10033601 GA and rs2292743 TA were independent risk factors for T2DM. According to the definition of genetics, the over-dominant model means the condition of a heterozygote having a phenotype that is more pronounced or better adapted than that of either homozygote. This may be due to the accumulation of different alleles. So heterozygotes for rs10033601 and rs2292743 were risk factors for the development of T2DM. Together, these results might provide convincing evidence assuming that genetic polymorphisms of the FBXW7 gene are associated with T2DM. However, the mechanisms that may link the FBXW7 genetic polymorphisms to T2DM remain unclear.

There are a number of limitations that might have influenced some of the study’s findings. First, this study was only carried out in the First Affiliated Hospital of the Xinjiang Medical University. Conclusions of the study were primarily drawn using the observed associations, and we failed to obtain a cause-and-effect relationship between the risk factors and T2DM. Second, the Uygur Chinese population is an admixed population that mainly lives in the Xinjiang Uygur Autonomous Region of China, and there is a lack of individual genetic background information. Third, the present study lacked a functional validation. Additional studies need to be undertaken to clarify the underlying molecular mechanisms that underlie the associations of the FBXW7 gene polymorphisms with T2DM. Four, The current research is only limited to the Uygur population, and the future work should try to supplement the research of Han and other ethnic groups as the control group.

## Conclusions

In conclusion, our study revealed that rs10033601 and rs2292743 of the FBXW7 gene were associated with T2DM in Uygur subjects in Xinjiang. Those who had the GA genotype of rs10033601 as well as those subjects who had the TA genotype of rs2292743 were found to have an association with the presence of T2DM.

## Data Availability

There will be no sharing of the obtained data since it will be required in another study.
